# GC-MS Fingerprinting Combined with Chemical Pattern-Recognition Analysis Reveals Novel Chemical Markers of the Medicinal Seahorse

**DOI:** 10.3390/molecules28237824

**Published:** 2023-11-28

**Authors:** Yuanyuan Jiang, Hongfei Wu, Paul Chi Lui Ho, Xuemei Tang, Hui Ao, Lu Chen, Jinjin Cai

**Affiliations:** 1 State Key Laboratory of Southwestern Chinese Medicine Resources, School of Pharmacy, Chengdu University of Traditional Chinese Medicine, Chengdu 611137, China; jiangyuanyuan@stu.edu.cn (Y.J.); wuhongfei@stu.cdutcm.edu.cn (H.W.); aohui2005@126.com (H.A.); 2 School of Pharmacy, Monash University Malaysia, Subang Jaya 47500, Malaysia; paul.ho@monash.edu; 3 Chengdu Institute of Food Inspection, Chengdu 610045, China; xmtang@stu.cdutcm.edu.cn; 4 Innovative Institute of Chinese Medicine and Pharmacy, Chengdu University of Traditional Chinese Medicine, Chengdu 611137, China; 5 Hospital of Chengdu University of Traditional Chinese Medicine, Chengdu 610075, China

**Keywords:** seahorse, *Hippocampus kelloggi*, *Hippocampus ingens*, GC-MS fingerprint, chemical pattern recognition

## Abstract

Seahorse is a valuable marine-animal drug widely used in traditional Chinese medicine (TCM), and which was first documented in the “Ben Cao Jing Ji Zhu” during the Liang Dynasty. *Hippocampus kelloggi* (HK) is the most common seahorse species in the medicinal material market and is one of the genuine sources of medicinal seahorse documented in the Chinese pharmacopeia. It is mainly cultivated in the Shandong, Fujian, and Guangxi Provinces in China. However, pseudo-HK, represented by *Hippocampus ingens* (HI) due to its similar appearance and traits, is often found in the market, compromising the safety and efficacy of clinical use. Currently, there is a lack of reliable methods for identifying these species based on their chemical composition. In this study, we employed, for the first time, a strategy combining gas chromatography-mass spectrometry (GC-MS) fingerprints and chemical patterns in order to identify HK and HI; it is also the first metabolomic study to date of HI as to chemical components. The obtained results revealed remarkable similarities in the chemical fingerprints, while significant differences were also observed. By employing hierarchical cluster analysis (HCA) and principal component analysis (PCA), based on the relative contents of their characteristic peaks, all 34 samples were successfully differentiated according to their species of origin, with samples from the same species forming distinct clusters. Moreover, nonadecanoic acid and behenic acid were exclusively detected in HK samples, further distinguishing them from HI samples. Additionally, the relative contents of lauric acid, tetradecanoic acid, pentadecanoic acid, n-hexadecanoic acid, palmitoleic acid, margaric acid, oleic acid, fenozan acid, eicosapentaenoic acid (EPA), and docosahexaenoic acid (DHA) exhibited significant differences between HK and HI (*p* < 0.0001), as determined by an unpaired *t*-test. Orthogonal partial least squares discriminant analysis (OPLS-DA) identified seven components (DHA, EPA, n-hexadecanoic acid, tetradecanoic acid, palmitoleic acid, octadecanoic acid, and margaric acid) with high discriminatory value (VIP value > 1). Thus, nonadecanoic acid, behenic acid, and these seven compounds can be utilized as chemical markers for distinguishing HK from HI. In conclusion, our study successfully developed a combined strategy of GC-MS fingerprinting and chemical pattern recognition for the identification of HK and HI, and we also discovered chemical markers that can directly differentiate between the two species. This study can provide a foundation for the authentication of Hippocampus and holds significant importance for the conservation of wild seahorse resources.

## 1. Introduction

**Figure 1:**
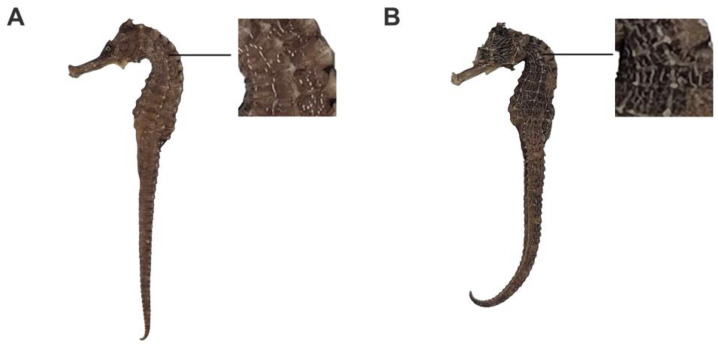
Photos of *H. kelloggi* (**A**) and *H. ingens* (**B**).

Seahorse is a valuable marine-animal drug widely used in traditional Chinese medicine (TCM). It was first documented in the “Ben Cao Jing Ji Zhu” during the Liang Dynasty and is revered for its kidney-warming, Yang-strengthening, knot-dispersing, and swelling-reducing properties, earning the title of “Animal Ginseng” [1]. The *Chinese Pharmacopoeia* (2020 edition) specifies five seahorse species as medicinal seahorse, including *Hippocampus kelloggi* Jordan et Snyder, *H. histrix* Kaup, *H. kuda* Bleeker, *H. trimaculatus* Leach, and *H. japonicas* Kaup [2]. However, our preliminary market survey and literature research have revealed a significant issue of species misidentification among medicinal seahorse, especially for *H. kelloggi* (HK). HK is the most common medical seahorse species in the traditional medicine market, and it is cultured in Shandong, Guangxi, Hainan, and other provinces of China [3,4]. It is called “Xian-wen seahorse” in Chinese, meaning “a seahorse with linear stripes”, due to its feature of intermittent small white dots on the head and trunk connected by vertical lines (Figure 1A). As it happens, another seahorse, the *H. ingens*, also possesses dense white vertical lines (Figure 1B) [5], making it visually similar to HK and leading to its fraudulent sale as HK in the market. Regrettably, HI is a unique seahorse species found in the Eastern Pacific Ocean and is not native to Chinese waters. It has not been documented in any traditional medicine literature, and there are no current studies on its chemical composition and pharmacological activity. Its risks and efficacy as a medicine are unclear. Furthermore, there are no reports on the artificial breeding of HI to date. It is quite possible that the HI appearing in the market is wild and mistaken for HK in international trade. The misidentification of medicinal seahorse not only weakens the safety and efficacy of treatments, but also exacerbates the threat to dwindling populations of wild seahorse.

Species authenticity plays a crucial role in ensuring the quality and clinical efficacy of traditional Chinese medicine (TCM), making it a significant focus in research on TCM resources. Compared with plant-based drugs, animal-derived drugs exhibit components that more closely resemble the human body, facilitating their absorption and therapeutic effects [6,7]. Hence, they are often referred to as “medicinal with lineage affinity to flesh and blood” in TCM, and known for their abundant resources, high activity, and remarkable healing properties. However, the diversity of species and the complexity of their composition, as well as challenges in species identification, pose a prominent issue of species confusion in animal-derived drugs, adversely affecting their efficacy and safety.

So far, the authenticity of seahorse has mainly been addressed through molecular techniques, such as DNA barcode-based identification using the *COI* gene [8,9,10]. However, this method is limited to the analysis of whole bodies, tissues, or powders from which DNA can be extracted, and it cannot be applied to extracts or preparations. Furthermore, DNA is chemically unstable and easily degraded, making it challenging to extract high-quality DNA from dried seahorse samples. Factors like temperature, light, and storage period can further impact the stability of DNA. Additionally, previous studies have indicated that the primary active components in Hippocampus are fatty acids, steroids, and other compounds [11]. DNA-based methods do not provide information on the identification of these active components, making them unsuitable for research establishing the effective substances, quality markers, and pharmacological activities of seahorse. Therefore, it is advisable to employ appropriate technical approaches to explore and discover an identification strategy for seahorse based on its chemical components.

The TCM fingerprint technique has gained international recognition as an effective method for identifying natural products, and is valued for its ability to provide a comprehensive representation of the overall chemical composition of TCM [12]. Gas chromatography-mass spectrometry (GC-MS) is an effective method for the separation, analysis, and identification of fatty acids, sterols, and other volatile components, with the advantages of high sensitivity, simple pre-treatment, low solvent-consumption, and good resolution, and it has been widely used in the study of the chemical composition of marine-animal drugs [11,13]. Chemical pattern recognition is a multivariate analysis technique such as hierarchical cluster analysis (HCA), principal component analysis (PCA), or orthogonal partial least squares discriminant analysis (OPLS-DA) which can reveal the rules behind measured data and has significant advantages in distinguishing samples through the analysis and visualization of high-dimensional data [14]. In recent years, the method of GC-MS fingerprinting combined with chemical pattern recognition has been effectively used in the identification, characterization, and quality evaluation of traditional medicines and foods rich in lipophilic components [15,16,17,18,19]. Therefore, it may be a viable approach to solve the aforementioned problem of medicinal seahorse.

In this study, the research strategy incorporating GC-MS fingerprints and chemical pattern recognition was employed, in response to the issue of HI seahorses being falsely sold as genuine HK seahorses, based on the characteristic chromatographic peaks. The differences in the characteristic components of HK and HI were detected for the first time through GC-MS fingerprinting. Subsequently, the HCA, PCA, and OPLS-DA techniques were applied to identify chemical markers that can directly differentiate between the two species. This study presents a novel method for authenticating medicinal seahorse and holds importance for the conservation of wild seahorse resources.

## 2. Results and Discussion

### 2.1. Fingerprints of H. kelloggi and H. ingens

**Figure 2:**
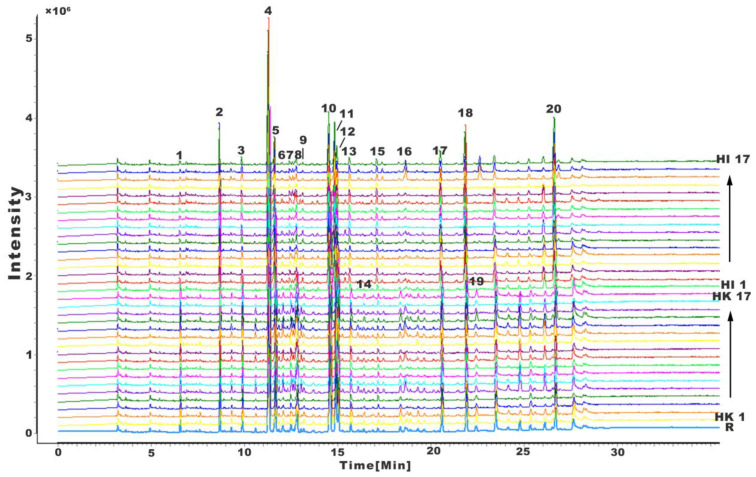
GC-MS chromatograms of HK and HI samples.

The GC-MS data for the 17 batches of HK were imported into the Chromatographic Fingerprint Evaluation System for Chinese Medicine (2012 version), and the width of the time window was set to 0.1 min. The peaks indicating relatively higher contents and distinct separations were selected for multi-point calibration using HK1 as the reference spectrum, the marked peaks were matched, and the median method was used to generate the control spectrum (R). The GC-MS data for the control spectrum (R) and the 34 batches of Hippocampus were then loaded into the software, as shown in Figure 2, and the control spectrum (R) was used as the reference spectrum. Likewise, a multi-point calibration was performed, and the marked-peak matching method was employed to calculate the similarity between the spectra of each batch of data and that of the control spectrum (R). As presented in Table 1, except for HI samples No. 1 and No. 5, the similarity value between all samples and the reference spectrum was above 0.930, indicating that the GC fingerprints of HK and HI were highly similar.

The chemical fingerprints revealed that HK demonstrated 20 shared characteristic peaks (peaks 1–20), while HI demonstrated 15 shared characteristic peaks (peaks 1, 2, 3, 4, 5, 7, 8, 10, 11, 12, 13, 15, 17, 18, and 20). The sums of these sets of peaks accounted for 87.22–93.76% (90.78 ± 2.06%) and 77.32–88.51% (84.08 ± 2.73%) of the total peak areas, respectively, indicating that they could effectively represent the chemical composition of the Hippocampus extract. There are a total of 15 common peaks in the fingerprints of HK and HI. These peaks were identified using the NIST14 database along with the literature, and the results are summarized in Table 2 and Table 3. The components with the highest relative content levels in both HK and HI were n-hexadecanoic acid, followed by octadecanoic acid, and then palmitoleic acid, elaidic acid, oleic acid, arachidonic acid, eicosapentaenoic acid (EPA), and docosahexaenoic acid (DHA). The relative content levels of these compounds in HK were 25.92 ± 1.29%, 14.67 ± 2.19%, 7.10 ± 1.25%, 7.13 ± 1.55%, 4.64 ± 0.90%, 4.21 ± 0.77%, and 4.53 ± 1.39%, respectively; those in HI were 22.38 ± 1.61%, 11.97 ± 1.84%, 4.87 ± 0.64%, 8.88 ± 1.15%, 3.22 ± 0.58%, 3.50 ± 0.91%, 9.16 ± 3.13%, and 10.04 ± 1.73%, respectively.

Although the chemical compositions of the two species were generally similar, as determined by total iron chromatography (TIC), there were obvious differences. The number of peaks from 16~30 min in HK was higher than that of HI. The two peaks with retention times of 16.422 and 22.494 min were detected in all the samples of HK but not in HI, and were identified as nonadecanoic acid and behenic acid. Despite their lower relative content levels of 0.27~0.58% and 0.61~1.22%, respectively, these two components can be used as important markers to distinguish HK from HI.

**Figure 3:**
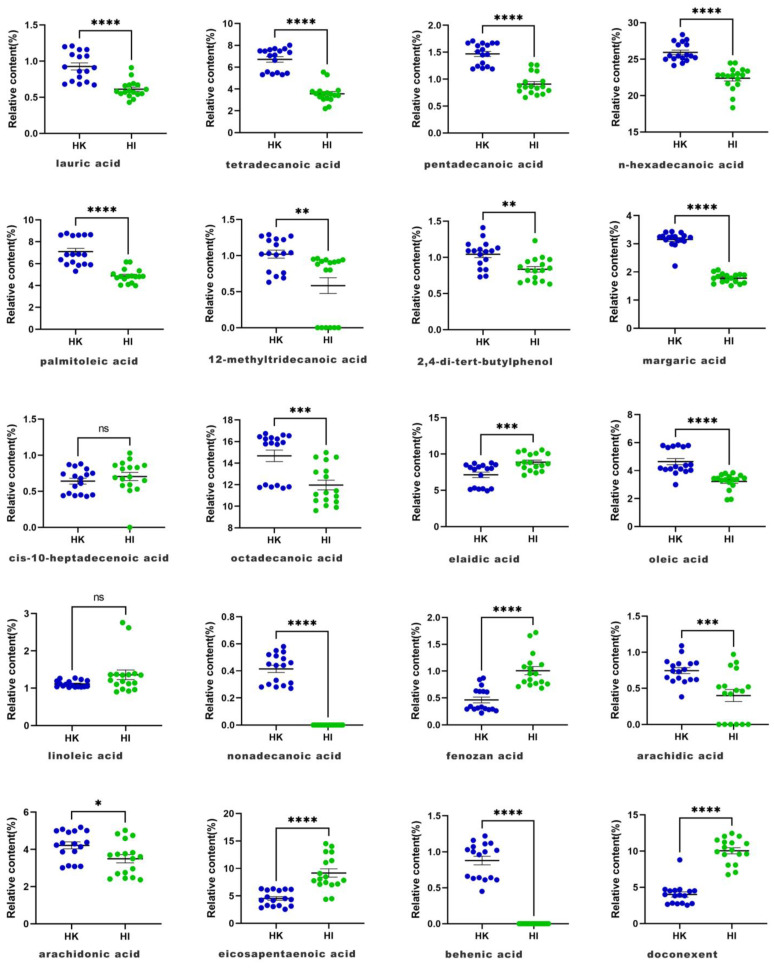
Relative content levels of the common peaks in total ion chromatography (TIC) of Hippocampus from two species; data are shown as mean ± SEM. **** *p* < 0.0001, *** *p* < 0.001, ** *p* < 0.01, * *p* < 0.05.

Additionally, an unpaired *t*-test was used to examine the relative content levels of the characteristic peaks of all the samples to identify the compounds which can be employed as index components for the comparison of HK and HI. The *p*-value was set as the filtering standard to maintain the contents. The results obtained revealed that the two species of Hippocampus differed significantly in their relative contents of a variety of compounds (Figure 3). The content levels of lauric acid, tetradecanoic acid, pentadecanoic acid, n-hexadecanoic acid, palmitoleic acid, margaric acid, and oleic acid in HK were significantly higher than those in HI (*p* < 0.0001), while the content levels of fenozan acid, EPA, and DHA in HI were significantly higher than those in HK (*p* < 0.0001). It is worth mentioning that in this study, Hippocampus was found to be rich in DHA and EDA. The relative content levels of DHA and EDA in HK extract were 4.21 ± 0.77% and 4.53 ± 1.39%, respectively, and their relative content levels in HI extract were 9.16 ± 3.13% and 10.04 ± 1.73%, respectively. The relative content levels of EPA and DHA were obviously higher in HI than in HK. EPA and DHA are both omega-3 polyunsaturated fatty acids. EPA can reduce blood viscosity, improve blood circulation, enhance tissue oxygenation, eliminate fatigue, and prevent atherosclerosis [20]. DHA, which is known as “brain gold”, is a major component of prostate glands and sperm, is crucial for cell growth, and is involved in the maintenance of the nervous system [21,22,23]. DHA and EPA are important active components of several marine drugs which play a nourishing role and demonstrate strong pharmacological effects. Although HI contains very high levels of DHA and EPA, it has not been included in any pharmacopeia, and its pharmacological activity and effectiveness deserve further study.

### 2.2. HCA

**Figure 4:**
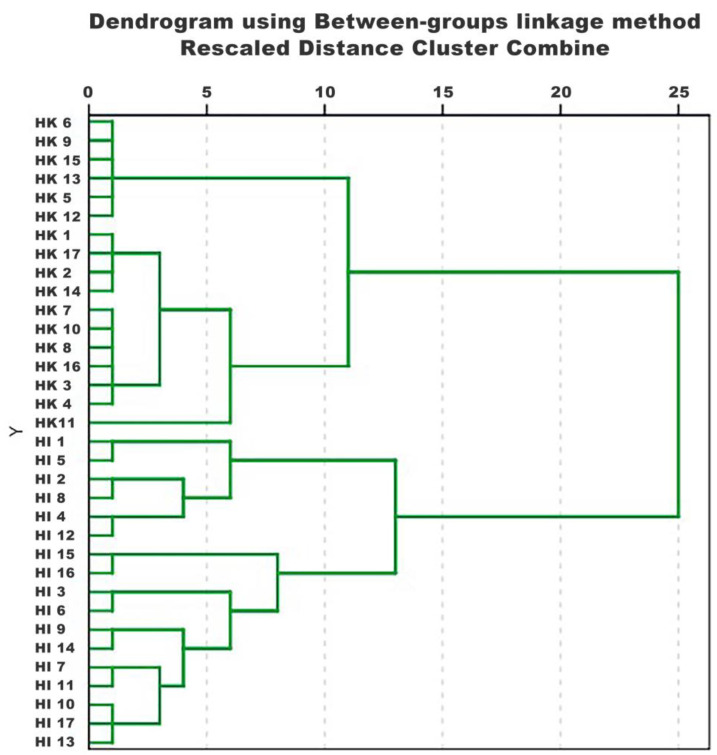
HCA dendrogram of 34 Hippocampus samples from the two species using the between-groups linkage method based on squared Euclidean distance.

HCA is a clustering method that assesses the degree of dissimilarity or similarity between the objects to be clustered. HCA will roughly group similar samples of Hippocampus into the same cluster based on the relative content levels of each component in the extracts. In order to find out the objective categories in the patterns of Hippocampus, the relative content levels of 20 characteristic components in the extracts were analyzed using HCA, with the parameter settings for “Between-groups linkage” and “Squared Euclidean distance”. As shown in Figure 4, when the classification distance was 25, 34 samples were divided into two categories. This demonstrated that HK and HI could be effectively distinguished by the HCA model based on those 20 characteristic components.

### 2.3. PCA

**Figure 5:**
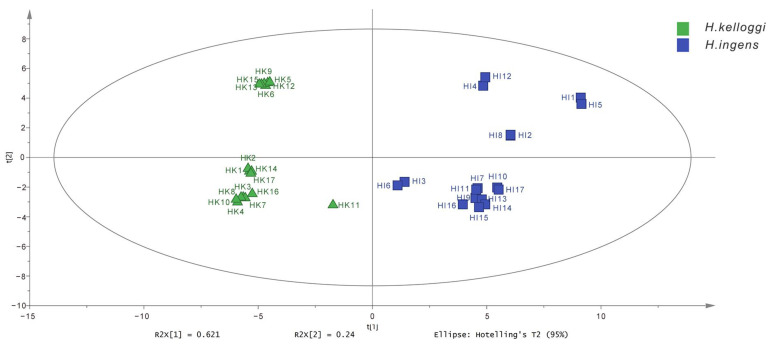
Score plot of principal component analysis of 34 samples of Hippocampus from two species.

PCA is an unsupervised analytical method used for the clustering and visualization of high-dimensional data through the application of dimensionality reduction and the extraction of several comprehensive indicators that can be used to explain the information obtained [24]. The first two principal components (PCs) explained the variability of approximately 86.1% of the original data. From the PCA scatter plot it can be observed that HK and HI clustered into two distinct regions, which was consistent with the results of the cluster analysis (Figure 5).

### 2.4. OPLS-DA

**Figure 6:**
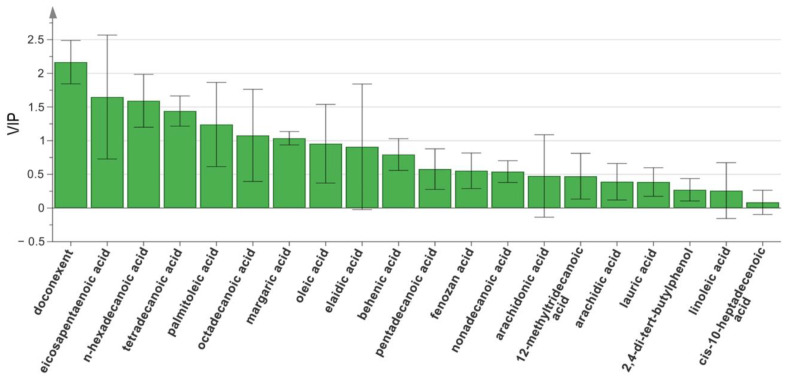
VIP values of the 20 characteristic components of 34 samples of Hippocampus from the two species, based on OPLS-DA.

Although HCA and PCA could clearly distinguish between HK and HI, neither could demonstrate the effects of variability on the classification of a sample. Thus, the relative content levels of 20 discrete components were analyzed using a supervised multivariate technique, OPLS-DA. This was to further elucidate the distinction between HK and HI and to identify the critical variables (key markers) that can be applied for the categorization of the samples into either of the two species. The contribution to the classification of the samples was positively correlated with the variable importance in the projection (VIP) value. In this study, a component with a VIP value > 1 was chosen as the primary chemical marker for the sample classification. Seven components with VIP values > 1, namely, DHA, EPA, n-hexadecanoic acid, tetradecanoic acid, palmitoleic acid, octadecanoic acid, and margaric acid were chosen as prospective markers (Figure 6).

In combination with the results of the previous unpaired *t*-test, six compounds—DHA, EPA, n-hexadecanoic acid, tetradecanoic acid, palmitoleic acid, and margaric acid—were found to possess significant (*p* < 0.0001) discriminative values. The relative content levels of DHA in all HK samples were <5%, except in HK11, in which they were >6% in all HI samples. The relative content levels of EPA were <7% in all HK samples, while they were >7% in almost all the HI samples, excepting HI15 and 16. The relative content levels of n-hexadecanoic acid were >24% in all HK samples, while they were <24% in almost all the HI samples, excepting HI3 and 6. The relative content levels of tetradecanoic acid were >5% in all the HK samples, whereas they were <5% in almost all the HI samples, excepting HI15 and 16. The relative content levels of palmitoleic acid were >5.8% in all the HK samples except HK11, while they were <5.5% in almost all the HI samples, excepting HI4 and 12. The relative content levels of margaric acid were >2.9% in all the HK samples except HK11, while they were <2.1% in all the HI samples.

## 3. Materials and Methods

### 3.1. Experimental Materials

#### 3.1.1. Samples

Hippocampus samples were collected from the Chengdu Hehuachi Chinese Herbal Medicine Market, Guangxi Yulin Chinese Herbal Medicine Market, and Guangzhou Qingping Chinese Herbal Medicine Market. A total of 34 batches, including 17 batches each of *H. kelloggi* and *H. ingens*, were collected and identified using a strategy of combined morphological identification and DNA barcoding. The information as to the 34 batches of samples is shown in Table 4.

#### 3.1.2. Reagents

Sodium hydroxide, hydrochloric acid, anhydrous ethanol, and anhydrous sodium sulfate, all of analytical grade, were purchased from the Chengdu Kolong Chemical Co., Ltd. (Chengdu, China); n-hexane and methanol, both of a chromatographically pure grade, were purchased from Thermo Fisher Scientific (China) Co., Ltd. (Shanghai, China).

#### 3.1.3. Instruments

Agilent 7890A-5975C GC–MS (7890A-5975C, Agilent, Santa Clara, CA, USA); HX-100 high-speed crusher (Xi’an Hardware and Pharmacy Tools Factory, Yongkang, Zhejiang, China); SQP type electronic analytical balance (Sartorius Scientific Instruments, Beijing, Co., Ltd., Beijing, China); KQ-300DE CNC ultrasonic cleaner (Kunshan Ultrasonic Instruments Co., Ltd., Shanghai, China); VM-500S vortex mixer (JOANLAB, Zhejiang, China).

### 3.2. Preparation of Sample Extracts

#### 3.2.1. Extraction of Samples

The different batches of seahorses were crushed separately to obtain a coarse powder. Then, 2 g of each batch of powder was accurately weighed and extracted with tenfold anhydrous ethanol as a solvent for 60 min by ultra sonification. The extracts were centrifuged for 10 min at 3000 rpm, and the supernatant was collected. The supernatant was concentrated, thereby obtaining the total extract.

#### 3.2.2. Methyl Esterification 

In accord with the relevant literature [25,26,27,28], an acid–base combined catalysis method was selected. Considering the different incubation times used in the literature, the incubation time was optimized. Nine different incubation times, namely, 0.5, 1, 2, 5, 10, 20, 30, 60, and 120 min, were set to examine the effect; the results are shown in Supplementary Figure S1 and Supplementary Tables S2 and S3. As can be seen, although the peak areas of fatty acid methyl ester increase with the extension of incubation time (Table S2), which reflects the more complete methyl esterification reaction, the final calculated relative content levels of fatty acids do not change much (Table S3), and there is little difference between the different incubation times. The similarities between these nine GC fingerprints (Figure S1) are all greater than 0.993 (Table 5), as evaluated by the “Chromatographic Fingerprint Similarity Evaluation System for Traditional Chinese Medicine” (2012 version). These findings indicate that the incubation time has little impact on the results of the research.

Based on the above research and the criterion of experimental efficiency, we chose the method of Jiang et al. [26] The sample extracts were methylated using the following method: The total extract was dissolved in n-hexane, and the same solvent was used to bring the final volume to 10 mL. Then, 2 mL was taken, and 1 mL of 2 mol/L NaOH-CH_3_OH was added for saponification, vortexed, and shaken for 10 min. Next, the solution was heated in a water bath at 50 °C for 5 min. The solution was cooled to room temperature, methyl esterified by adding 2 mL of 2 mol/L HCl-CH_3_OH, vortexed and shaken for 10 min, and then heated for 5 min in a water bath at 50 °C. The supernatant was washed with 2mL of distilled water, the aqueous layer was removed, and, after dehydration by adding anhydrous sodium sulfate, the supernatant was collected and filtered through 0.22 μm sized microporous membranes for GC-MS analysis.

### 3.3. GC-MS Analysis

The chromatographic conditions were set as follows—HP-5 MS (Agilent, Santa Clara, CA, USA, 30 m × 0.25 mm × 0.25 µm); carrier gas: He (99.999% purity); flow rate: 1 mL/min; inlet temperature: 250 °C; detector temperature: 250 °C; split ratio: 10:1; column temperature: programmed ramp rate of 100~180 °C, 10 °C/min, 180~250 °C, 3.3 °C/min, and kept for 20 min; and sample volume: 1 μL.

The mass spectrometry conditions were set as—EI source: electron energy 70 eV; ionization temperature: 150 °C; doubling voltage: 1576 V; interface temperature: 280 °C; mass range: 10~550 amu; and solvent delay time: 3 min.

The compounds were identified through the NIST14 mass spectrometry database (National Institute of Standards and Technology). The area normalization method was used to calculate the relative content of each compound in the chromatogram.

### 3.4. Methodological Examination

#### 3.4.1. Precision Test

The samples were taken for analysis in five successive injections. The similarities in each spectrum were >0.99 using the precision test. The relative retention time RSD of the 15 major common peaks was <0.01% and the peak area RSD was 0.02~0.16%, indicating a high precision of the instrument used.

#### 3.4.2. Stability Test

The samples were analyzed at six time points of 0, 2, 4, 8, 16, and 24 h. The similarities between all the obtained spectra were >0.99 from the stability test. The relative retention time RSD of the 15 major common peaks was <0.01%, and the peak area RSD range from 0.13~0.27%, indicating that the sample solution was stable for at least 24 h.

#### 3.4.3. Repeatability Test

Five samples from the same batch of seahorses were extracted and analyzed separately. The similarity in the obtained spectra of each batch was >0.99, as determined by the repeatability test. The relative retention time RSD of the 15 major common peaks was <0.01%, and the peak area RSD was 0.06~0.25%, indicating that the method used was highly reproducible.

### 3.5. Data Analysis

The similarity between the GC fingerprints was evaluated using the “Chromatographic Fingerprint Similarity Evaluation System for Traditional Chinese Medicine” (2012 version). The statistical analyses using an unpaired *t*-test were carried out employing GraphPad Prism 9 (GraphPad Software Inc., La Jolla, CA, USA). Moreover, these data were also analyzed using the HCA, PCA, and OPLS-DA, employing SPSS26.0 (SPSS Inc., Chicago, IL, USA) or SIMCA-P14.1, (Umetrics, Umea, Sweden). The results are expressed as mean values ± standard error of the mean (SEM) and a *p* < 0.05 was considered to be statistically significant.

## 4. Conclusions

In summary, this study established an effective strategy combining GC-MS fingerprints and chemical pattern recognition to identify genuine HK seahorse and its counterfeit, HI seahorse, for the first time, and further found chemical markers that can directly be used for the identification. The GC-MS fingerprints showed that there were 15 peaks common to HK and HI. Although the fingerprints of HK and HI were similar, significant differences were also observed. The comprehensive analysis utilizing GC-MS fingerprint, HCA, and PCA demonstrated a clear distinction between HK and HI samples. Notably, nine compounds, nonadecanoic acid, behenic acid, DHA, EPA, n-hexadecanoic acid, tetradecanoic acid, palmitoleic acid, octadecanoic acid, and margaric acid, were identified as chemical markers crucial for distinguishing HK from HI. These findings emphasize the significance of species origin in determining the quality of TCM. This study provides a scientific and technical foundation for the explicit authentication of Hippocampus, paves the way for further research on its chemical composition, and contributes to the conservation of wild natural resources.

## Figures and Tables

**Table 1:** Results of the similarity evaluation between the mean HK profiles and the profiles of 34 batches of Hippocampus samples.

Similarity	Sample No.																
	1	2	3	4	5	6	7	8	9	10	11	12	13	14	15	16	17
HK	0.997	0.997	0.992	0.990	0.995	0.996	0.992	0.992	0.994	0.993	0.979	0.994	0.994	0.996	0.994	0.990	0.996
HI	0.885	0.937	0.967	0.951	0.879	0.968	0.945	0.940	0.941	0.935	0.938	0.946	0.933	0.936	0.931	0.937	0.937

**Table 2:** The relative content levels of the peaks common to the two species in HK.

No.	Retention Time	Component	Molecular Formula	Relative Content (%)																	
				1	2	3	4	5	6	7	8	9	10	11	12	13	14	15	16	17	Mean ± S.D.
1	6.567	lauric acid	C_12_H_24_O_2_	0.91	0.86	1.07	1.06	0.78	0.68	1.16	1.2	0.68	1.21	1.09	0.71	0.67	0.86	0.72	1.16	0.92	0.93 ± 0.20
2	8.677	tetradecanoic acid	C_12_H_24_O_2_	7.06	7.48	5.32	5.35	7.47	7.6	5.49	5.44	7.69	5.57	6.64	7.49	7.7	7.35	8.01	5.32	7.11	6.71 ± 1.03
3	9.889	pentadecanoic acid	C_14_H_28_O_2_	1.55	1.46	1.63	1.62	1.19	1.23	1.71	1.67	1.19	1.67	1.67	1.2	1.24	1.51	1.3	1.67	1.45	1.47 ± 0.20
4	11.303	n-hexadecanoic acid	C_15_H_30_O_2_	25.44	25.57	28.36	26.98	24.12	24.82	27.44	27.69	25.06	27.56	25.47	24.43	25.16	25.21	24.98	26.97	25.44	25.92 ± 1.29
5	11.64	palmitoleic acid	C_16_H_32_O_2_	6.82	6.84	5.92	5.9	8.57	8.61	5.96	6.14	8.62	6.36	5.31	8.65	8.64	6.86	8.77	5.85	6.81	7.10 ± 1.25
6	12.055	12-methyltridecanoic acid	C_16_H_30_O_2_	0.76	0.71	1.04	1.15	1.29	1.27	1.01	1.03	1.22	1.03	0.63	1.23	1.27	0.69	1.21	1.02	0.77	1.02 ± 0.23
7	12.482	2,4-di-tert-butylphenol	C_14_H_28_O_2_	0.83	0.74	1.11	1.41	1.13	1.08	0.97	0.92	1.09	1.18	1.18	1.09	1.09	0.73	1.04	1.3	0.83	1.04 ± 0.19
8	12.819	margaric acid	C_14_H_22_O	3.09	2.96	3.28	3.41	3.25	3.13	3.26	3.43	3.22	3.4	2.21	3.14	3.22	2.99	3.11	3.24	3.24	3.15 ± 0.28
9	13.144	cis-10-heptadecenoic acid	C_17_H_34_O_2_	0.57	0.71	0.85	0.73	0.45	0.43	0.61	0.88	0.44	0.87	0.74	0.47	0.45	0.68	0.44	0.75	0.81	0.64 ± 0.17
10	14.569	octadecanoic acid	C_17_H_32_O_2_	16.45	15.92	15.78	16.61	11.75	11.94	16.36	15.87	11.79	16.25	16.54	11.81	11.68	16.24	11.99	15.74	16.74	14.67 ± 2.19
11	14.884	elaidic acid	C_18_H_36_O_2_	8.01	7.89	8.52	8.15	5.16	5.31	8.49	8.77	5.12	8.71	7.07	5.19	4.93	7.98	5.21	8.43	8.23	7.13 ± 1.55
12	15.007	oleic acid	C_18_H_34_O_2_	4.37	4.12	4.02	3.82	5.7	5.79	4.11	4.28	5.79	3.94	2.99	5.74	5.65	4.17	5.83	4.08	4.42	4.64 ± 0.90
13	15.681	linoleic acid	C_18_H_34_O_2_	1.04	1.06	1.2	1.17	1.07	1.04	1.19	1.26	1.05	1.27	1.12	1.1	1.03	1.02	1.03	1.23	1.04	1.11 ± 0.09
14	16.422	nonadecanoic acid	C_18_H_32_O_2_	0.44	0.44	0.51	0.58	0.31	0.3	0.49	0.54	0.28	0.55	0.33	0.29	0.28	0.45	0.27	0.52	0.46	0.41 ± 0.11
15	17.151	fenozan acid	C_18_H_32_O_2_	0.34	0.29	0.61	0.84	0.29	0.31	0.62	0.63	0.3	0.62	0.74	0.28	0.29	0.26	0.22	0.87	0.33	0.46 ± 0.22
16	18.341	arachidic acid	C_17_H_26_O_3_	0.84	0.84	0.73	1.09	0.62	0.62	0.76	0.74	0.59	0.81	0.38	0.6	0.65	0.87	0.64	1.01	0.85	0.74 ± 0.17
17	20.574	arachidonic acid	C_20_H_36_O_2_	3.12	3.01	4.38	4.14	4.92	5.08	4.34	3.89	5.19	4.3	3.87	4.99	5	3.09	5.01	4.23	3.08	4.21 ± 0.77
18	21.91	eicosapentaenoic acid	C_20_H_32_O_2_	4.32	4.2	3.27	2.55	5.98	6.22	3.22	3.04	6.28	2.82	4.79	6.29	6.06	4.16	6.17	3.13	4.5	4.53 ± 1.39
19	22.494	behenic acid	C_20_H_30_O_2_	0.96	1.02	1.11	1.22	0.64	0.66	1	1	0.64	1.12	0.45	0.61	0.61	1.07	0.63	1.16	1.03	0.88 ± 0.25
20	26.691	docosahexaenoic acid	C_24_H_48_O_2_	3.84	3.83	4.7	4.5	2.53	2.76	4.71	4.44	2.8	4.52	8.79	2.66	2.81	4	2.72	4.57	3.93	4.00 ± 1.48
Total percentage of common compounds				90.76	89.95	93.41	92.28	87.22	88.88	92.9	92.86	89.04	93.76	92.01	87.97	88.43	90.19	89.3	92.25	91.99	90.78 ± 2.06

**Table 3:** The relative content levels of the peaks common to the two species in HI.

No.	Retention Time	Component	Molecular Formula	Relative Content (%)																	
				1	2	3	4	5	6	7	8	9	10	11	12	13	14	15	16	17	Mean ± S.D.
1	6.567	lauric acid	C_12_H_24_O_2_	0.59	0.43	0.66	0.67	0.67	0.57	0.47	0.55	0.81	0.69	0.57	0.61	0.58	0.91	0.55	0.54	0.52	0.61 ± 0.12
2	8.677	tetradecanoic acid	C_12_H_24_O_2_	3.54	3.29	3.07	3.58	3.4	3.05	3.81	3.14	2.2	3.65	3.78	3.53	3.43	2.36	5.31	5.55	3.88	3.56 ± 0.84
3	9.889	pentadecanoic acid	C_14_H_28_O_2_	0.83	0.88	0.85	0.79	0.7	0.86	1.15	0.78	0.86	0.96	1.17	0.72	0.75	0.66	1.27	1.26	0.95	0.91 ± 0.19
4	11.303	n-hexadecanoic acid	C_15_H_30_O_2_	18.33	22.83	24.46	22.81	19.49	24.48	23.02	22.67	21.63	22.84	23.35	22.79	23.52	21.53	20.95	22.29	23.55	22.38 ± 1.61
5	11.64	palmitoleic acid	C_16_H_32_O_2_	4.7	5.06	5.34	6.15	4.84	5.48	4.81	4.87	4.09	4.61	4.83	6.15	4.84	3.99	4.03	4.23	4.8	4.87 ± 0.64
6	12.055	12-methyltridecanoic acid	C_16_H_30_O_2_	0.94	0.88	0.94	0.95	0.92	0.96	0.9	0.92	0	0.8	0.8	0.91	0	0	0	0	0	0.58 ± 0.45
7	12.482	2,4-di-tert-butylphenol	C_14_H_28_O_2_	1.23	0.67	0.82	1	0.94	0.85	0.63	0.65	0.97	0.68	0.65	0.97	0.9	0.87	0.81	0.82	0.72	0.83 ± 0.16
8	12.819	margaric acid	C_14_H_22_O	1.77	1.56	1.93	2.07	1.84	1.91	1.65	1.57	1.89	1.78	1.51	2.02	1.87	1.68	1.61	1.66	1.88	1.78 ± 0.17
9	13.144	cis-10-heptadecenoic acid	C_17_H_34_O_2_	0.58	0.81	0.83	0.53	0.59	0.83	1.03	0.86	0	0.65	0.95	0.51	0.86	0.89	0.69	0.7	0.67	0.70 ± 0.24
10	14.569	octadecanoic acid	C_17_H_32_O_2_	10.61	10.93	14.56	10.42	11.11	14.98	11.43	10.53	14.32	12.74	11.26	9.6	13.26	14.57	10.06	9.9	13.17	11.97 ± 1.84
11	14.884	elaidic acid	C_18_H_36_O_2_	7.61	8.87	8.26	7.41	7.7	8.13	10.56	8.66	9.91	8.62	10.29	7.07	8.42	10.48	10.09	10.05	8.82	8.88 ± 1.15
12	15.007	oleic acid	C_18_H_34_O_2_	3.44	3.46	3.71	3.84	3.36	3.8	3.57	3.35	2.95	3.3	3.34	3.65	2.58	3.16	1.94	1.91	3.43	3.22 ± 0.58
13	15.681	linoleic acid	C_18_H_34_O_2_	0.97	0.96	1.14	1.21	0.9	1.18	1.35	0.92	2.62	1.37	1.35	1.12	1.1	2.76	1.36	1.39	1.36	1.36 ± 0.53
14	16.422	nonadecanoic acid	C_18_H_32_O_2_	0	0	0	0	0	0	0	0	0	0	0	0	0	0	0	0	0	-
15	17.151	fenozan acid	C_18_H_32_O_2_	0.71	0.74	1.12	0.8	0.68	1.08	0.8	0.76	1.72	0.95	0.84	0.77	1.33	1.66	1.05	1.15	0.95	1.01 ± 0.31
16	18.341	arachidic acid	C_17_H_26_O_3_	0.52	0.78	0.97	0.43	0.53	0	0	0.46	0	0.52	0	0.42	0.86	0	0.47	0	0.82	0.40 ± 0.34
17	20.574	arachidonic acid	C_20_H_36_O_2_	3.61	3.83	2.48	5.02	3.74	2.37	4.59	3.77	3.5	2.75	4.75	4.84	2.69	3.7	2.41	2.45	2.96	3.50 ± 0.91
18	21.91	eicosapentaenoic acid	C_20_H_32_O_2_	14.01	11.42	7.44	12.99	14.51	7.1	7.16	11.06	7.83	8.46	7.01	13.06	7.75	8.04	4.36	4.49	9.07	9.16 ± 3.13
19	22.494	behenic acid	C_20_H_30_O_2_	0	0	0	0	0	0	0	0	0	0	0	0	0	0	0	0	0	-
20	26.691	docosahexaenoic acid	C_24_H_48_O_2_	9.91	9.62	8.06	7.05	10.51	8.08	11.03	9.52	9.57	12.05	11.22	6.75	12.03	10.14	11.52	11.14	12.45	10.04 ± 1.73
Total percentage of common compounds				83.9	87.02	86.64	87.72	86.43	85.71	87.96	85.04	84.87	87.42	87.67	85.49	86.77	87.4	78.48	79.53	90	85.77 ± 2.92

**Table 4:** Information as to the 34 batches of hippocampal samples.

No.	Species	Collection Place	Batch Number	No.	Species	Collection Place	Batch Number
HK1	*H. kelloggi*	Chengdu Hehuachi Traditional Chinese Medicine Market	HK20230301H	HI1	*H. ingens*	Chengdu Hehuachi Traditional Chinese Medicine Market	HI20220901H
HK2	*H. kelloggi*	Chengdu Hehuachi Traditional Chinese Medicine Market	HK20230302H	HI2	*H. ingens*	Chengdu Hehuachi Traditional Chinese Medicine Market	HI20220902H
HK3	*H. kelloggi*	Chengdu Hehuachi Traditional Chinese Medicine Market	HK20230303H	HI3	*H. ingens*	Chengdu Hehuachi Traditional Chinese Medicine Market	HI20220903H
HK4	*H. kelloggi*	Chengdu Hehuachi Traditional Chinese Medicine Market	HK20230304H	HI4	*H. ingens*	Chengdu Hehuachi Traditional Chinese Medicine Market	HI20220904H
HK5	*H. kelloggi*	Chengdu Hehuachi Traditional Chinese Medicine Market	HK20230305H	HI5	*H. ingens*	Chengdu Hehuachi Traditional Chinese Medicine Market	HI20221105H
HK6	*H. kelloggi*	Chengdu Hehuachi Traditional Chinese Medicine Market	HK20230306H	HI6	*H. ingens*	Chengdu Hehuachi Traditional Chinese Medicine Market	HI20221106H
HK7	*H. kelloggi*	Chengdu Hehuachi Traditional Chinese Medicine Market	HK20230307H	HI7	*H. ingens*	Guangxi Yulin Traditional Chinese Medicine Market	HI20221107Q
HK8	*H. kelloggi*	Chengdu Hehuachi Traditional Chinese Medicine Market	HK20230308H	HI8	*H. ingens*	Guangxi Yulin Traditional Chinese Medicine Market	HI20221108Y
HK9	*H. kelloggi*	Guangxi Yulin Traditional Chinese Medicine Market	HK20221101Y	HI9	*H. ingens*	Guangxi Yulin Traditional Chinese Medicine Market	HI20221109Y
HK10	*H. kelloggi*	Guangxi Yulin Traditional Chinese Medicine Market	HK20221102Y	HI10	*H. ingens*	Guangxi Yulin Traditional Chinese Medicine Market	HI20221110Y
HK11	*H. kelloggi*	Guangxi Yulin Traditional Chinese Medicine Market	HK 20221103Y	HI11	*H. ingens*	Guangxi Yulin Traditional Chinese Medicine Market	HI20221111Y
HK12	*H. kelloggi*	Guangxi Yulin Traditional Chinese Medicine Market	HK20221104Y	HI12	*H. ingens*	Guangxi Yulin Traditional Chinese Medicine Market	HI20221112Y
HK13	*H. kelloggi*	Guangxi Yulin Traditional Chinese Medicine Market	HK20221105Y	HI13	*H. ingens*	Guangzhou Qingping Traditional Chinese Medicine Market	HI20221113Q
HK14	*H. kelloggi*	Guangzhou Qingping Traditional Chinese Medicine Market	HK20221106Q	HI14	*H. ingens*	Guangzhou Qingping Traditional Chinese Medicine Market	HI20221114Q
HK15	*H. kelloggi*	Guangzhou Qingping Traditional Chinese Medicine Market	HK20221107Q	HI15	*H. ingens*	Guangzhou Qingping Traditional Chinese Medicine Market	HI20221115Q
HK16	*H. kelloggi*	Guangzhou Qingping Traditional Chinese Medicine Market	HK20221108H	HI16	*H. ingens*	Guangzhou Qingping Traditional Chinese Medicine Market	HI20221116Q
HK17	*H. kelloggi*	Guangzhou Qingping Traditional Chinese Medicine Market	HK20221109H	HI17	*H. ingens*	Guangzhou Qingping Traditional Chinese Medicine Market	H20221117Q

**Table 5:** Similarity of GC fingerprints for nine different incubation times.

Incubation Time (min)	0.5	1	2	5	10	20	30	60	120
0.5	1	1	1	1	0.999	0.999	0.998	0.998	0.993
1	1	1	1	1	0.999	0.999	0.998	0.998	0.994
2	1	1	1	1	0.999	0.999	0.999	0.998	0.994
5	1	1	1	1	1	0.999	0.999	0.998	0.994
10	0.999	0.999	0.999	1	1	1	1	0.999	0.996
20	0.999	0.999	0.999	0.999	1	1	0.999	0.999	0.996
30	0.998	0.998	0.999	0.999	1	0.999	1	1	0.997
60	0.998	0.998	0.998	0.998	0.999	0.999	1	1	0.998
120	0.993	0.994	0.994	0.994	0.996	0.996	0.997	0.998	1

## Data Availability

All relevant data are contained within the article.

## Supplementary Materials

The following supporting information can be downloaded at: https://www.mdpi.com/article/10.3390/molecules28237824/s1, Figure S1: GC fingerprints of seahorse extract for 9 different incubation times; Table S1: Peak areas of main components for 9 different incubation times; Table S2: Relative content (%) of main components for 9 different incubation times.
