# Supplementary material for: GC-MS Fingerprinting Combined with Chemical Pattern-Recognition Analysis Reveals Novel Chemical Markers of the Medicinal Seahorse
Source: Molecules. 2023 Nov 28;28(23):7824. doi: 10.3390/molecules28237824 (PMC10708380; doi:10.3390/molecules28237824)
Supplement: Supplementary file 1 [file molecules-28-07824-s001.zip › molecules-2664748-supplementary.pdf]

Table S1. Peak areas of main components for 9 different incubation times.

No.	Retention Time	Component	Peak Area								
			0.5min	1min	2min	5min	10min	20min	30min	60min	120
1	6.320	methyl laurate	645350	529586	686910	818932	964060	1254681	1482307	1707511	2654681
2	8.363	methyl tetradecanoate	9690699	9256389	10352933	9549470	9178340	19956017	12648033	13739551	18629667
3	9.519	methyl pentadecanoate	1924134	2136321	2178214	2007276	2414418	4884670	2784450	2885592	3978540
4	10.843	methyl hexadecanoate	42221388	40402057	45444019	42433709	42618131	94480667	63040908	71389843	108174575
	22.516	n-hexadecanoic acid	12440551	12020839	9900611	10670454	10069736	13770165	5908517	3692989	1655163
		methyl hexadecanoate + n-hexadecanoic acid	54661939	52422896	55344630	53104163	52687867	108250832	68949425	75082832	109829738
5	11.157	methyl palmitoleate	9742728	9158303	9790751	9453857	9342234	19931855	12867240	14331684	19507887
6	11.864	2,4-Di-tert-butylphenol	2265346	1988241	2052772	2463125	2512893	4549372	3371435	3391209	6208333
7	12.302	methyl margarate	3016549	2899912	3109540	2965587	2868861	6345431	3921760	4342845	6456922
8	13.975	methyl stearate	13696241	13027893	14774804	14045673	14997866	31867130	23389564	26951090	46151009
	26.635	octadecanoic acid	7125829	5816322	4230809	5469759	5104558	7888532	4035119	2447003	1139269
		methyl stearate + octadecanoic acid	20822070	18844215	19005613	19515432	20102424	39755662	27424683	29398093	47290278
9	14.266	methyl trans-9-octadecenoate	19422029	18604914	21215246	19355772	19082643	42108253	28329682	31344327	45836902
10	14.379	methyl cis-9-octadecenoate	3876600	3844893	4023735	3717733	3593349	8454141	5756896	6036562	9331756
	27.151	oleic acid	5251024	5363789	3435212	6725513	4765266	10758038	3778151	999895	0
		methyl cis-9-octadecenoate + oleic acid	9127624	9208682	7458947	10443246	8358615	19212179	9535047	7036457	9331756
11	15.018	methyl octadiene-9,12-dienecarboxylate	4795514	4561522	4562288	4281324	4142321	8353240	6302196	6045676	8769512
12	16.388	methyl 3-(3,5-Di-tert-butyl-4-hydroxyphenyl) propionate	620812	581849	523381	884367	1522399	1613690	2526114	2612015	3254046
13	19.755	methyl arachidonate	2791210	2814896	2966729	3192000	2893830	6921844	4591202	5330233	7386349
14	21.034	methyl cis-5,8,11,14,17-eicosapentaenoate	5395139	5369979	5999213	5713119	5497889	11410231	8298579	8974083	12213698
15	25.748	methyl 4,7,10,13,16,19-docosahexaenoate	14518373	13270048	15490931	14794930	14362182	29685345	22329019	24380381	33277152

Table S2. Relative content (%) of main components for 9 different incubation times.

No.	Retention Time	Component		Relative content (%)									
		Before methylation	After methylation	0.5min	1min	2min	5min	10min	20min	30min	60min	120min	mean±S.D
1	6.320	lauric acid	methyl laurate	0.3	0.27	0.33	0.4	0.48	0.29	0.53	0.57	0.6	0.42±0.13
2	8.363	tetradecanoic acid	methyl tetradecanoate	4.54	4.68	4.91	4.68	4.6	4.66	4.55	4.59	4.18	4.60±0.19
3	9.519	pentadecanoic acid	methyl pentadecanoate	0.9	1.08	1.03	0.98	1.21	1.14	1	0.96	0.89	1.02±0.11
4	10.843	n-hexadecanoic acid	methyl hexadecanoate	19.79	20.42	21.54	20.79	21.36	22.05	22.66	23.84	24.27	21.86±1.51
	22.516		n-hexadecanoic acid	5.83	6.08	4.69	5.23	5.05	3.21	2.12	1.23	0.37	3.76±2.10
	—		methyl hexadecanoate + n-hexadecanoic acid	25.62	26.5	26.23	26.02	26.41	25.26	24.78	25.07	24.64	25.61±0.71
5	11.157	palmitoleic acid	methyl palmitoleate	4.57	4.63	4.64	4.63	4.68	4.65	4.63	4.79	4.38	4.62±0.11
6	11.864	2,4-di-tert-butylphenol	2,4-Di-tert-butylphenol	1.06	1.01	0.97	1.21	1.26	1.06	1.21	1.13	1.39	1.14±0.13
7	12.302	margaric acid	methyl margarate	1.41	1.47	1.47	1.45	1.44	1.48	1.41	1.45	1.45	1.45±0.02
8	13.975	octadecanoic acid	methyl stearate	6.42	6.59	7	6.88	7.52	7.44	8.41	9	10.35	7.73±1.29
	26.635		octadecanoic acid	3.34	2.94	2	2.68	2.56	1.84	1.45	0.82	0.26	1.99±1.01
	—		methyl stearate + octadecanoic acid	9.76	9.53	9	9.56	10.08	9.28	9.86	9.82	10.61	9.72±0.46
9	14.266	elaidic acid	methyl trans-9-octadecenoate	9.1	9.4	10.05	9.48	9.56	9.83	10.18	10.47	10.28	9.82±0.46
10	14.379	oleic acid	methyl cis-9-octadecenoate	1.82	1.94	1.91	1.82	1.8	1.97	2.07	2.02	2.09	1.94±0.11
	27.151		oleic acid	2.46	2.71	1.63	3.3	2.39	2.51	1.36	0.33	0	1.85±1.12
	—		methyl cis-9-octadecenoate + oleic acid	4.28	4.65	3.54	5.12	4.19	4.48	3.43	2.35	2.09	3.79±1.03
11	15.018	linoleic acid	methyl octadiene-9,12-dienecarboxylate	2.25	2.31	2.16	2.1	2.08	1.95	2.27	2.02	1.97	2.12±0.13
12	16.388	fenozan acid	methyl 3-(3,5-di-Tert-Butyl-4-hydroxyphenyl) propionate	0.29	0.29	0.25	0.43	0.76	0.38	0.91	0.87	0.73	0.55±0.27
13	19.755	arachidonic acid	methyl arachidonate	1.31	1.42	1.41	1.56	1.45	1.62	1.65	1.78	1.66	1.54±0.15
14	21.034	eicosapentaenoic acid	methyl cis-5,8,11,14,17-eicosapentaenoate	2.53	2.71	2.84	2.8	2.76	2.66	2.98	3	2.74	2.78±0.15
15	25.748	docosahexaenoic acid	methyl 4,7,10,13,16,19-docosahexaenoate	6.81	6.71	7.34	7.25	7.2	6.93	8.03	8.14	7.47	7.32±0.50

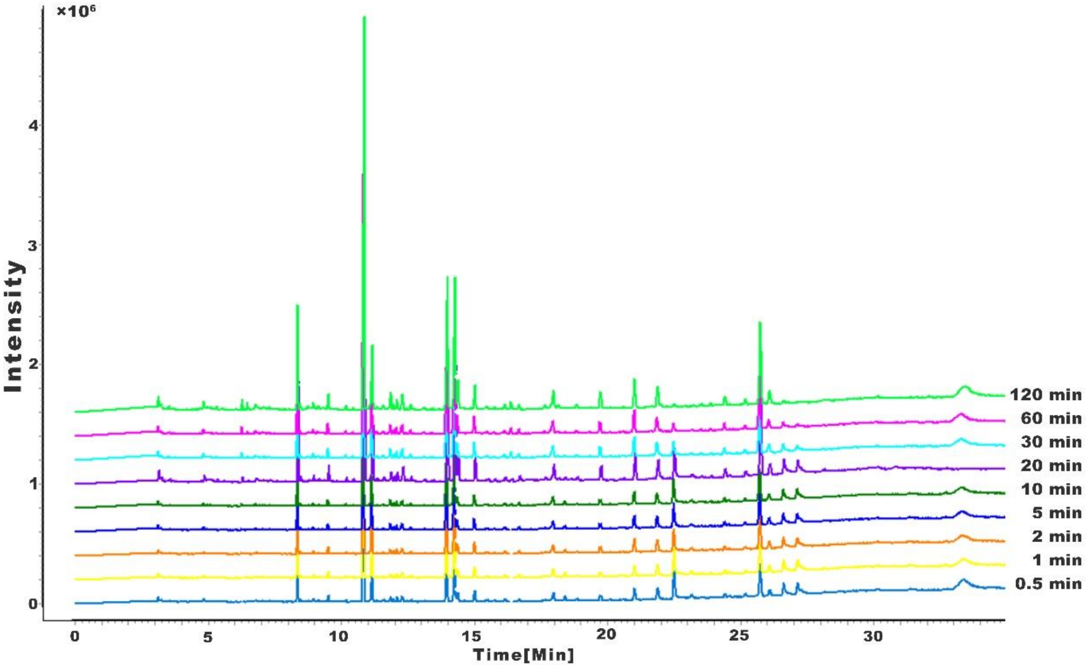

Figure S1. GC fingerprints of seahorse extract for 9 different incubation times.
